# Supplementary material for: OpenMM 7: Rapid development of high performance algorithms for molecular dynamics
Source: PLoS Comput Biol. 2017 Jul 26;13(7):e1005659. doi: 10.1371/journal.pcbi.1005659 (PMC5549999; doi:10.1371/journal.pcbi.1005659)
Supplement: S1 Source Code — (ZIP) [file pcbi.1005659.s002.zip › OpenMM-7.0.1-Source/docs/api-c++/index.html]

OpenMM C++ API — OpenMM @OPENMM\_MAJOR\_VERSION@.@OPENMM\_MINOR\_VERSION@ documentation


# OpenMM C++ API¶

The C++ API provides information about the classes and methods available in OpenMM for C++ developers. The public API is based on a small number of classes.

- Core Objects
- Forces
- Integrators
- Extras

`System`: A System specifies generic properties of the system to be
simulated: the number of particles it contains, the mass of each one, the size
of the periodic box, etc. The interactions between the particles are specified
through a set of Force objects (see below) that are added to the System. Force
field specific parameters, such as particle charges, are not direct properties
of the System. They are properties of the Force objects contained within the
System.

`Force`: The Force objects added to a System define the behavior of the
particles. Force is an abstract class; subclasses implement specific behaviors.
The Force class is actually slightly more general than its name suggests. A
Force can, indeed, apply forces to particles, but it can also directly modify
particle positions and velocities in arbitrary ways. Some thermostats and
barostats, for example, can be implemented as Force classes. Examples of Force
subclasses include `HarmonincBondForce`, `NonbondedForce`, and `MonteCarloBarostat`.

`Context`: This stores all of the state information for a simulation:
particle positions and velocities, as well as arbitrary parameters defined by
the Forces in the System. It is possible to create multiple Contexts for a
single System, and thus have multiple simulations of that System in progress at
the same time.

`Integrator`: This implements an algorithm for advancing the simulation
through time. It is an abstract class; subclasses implement specific
algorithms. Examples of Integrator subclasses include `LangevinIntegrator`,
`VerletIntegrator`, and `BrownianIntegrator`.

`State`: A State stores a snapshot of the simulation at a particular point
in time. It is created by calling a method on a Context. This is the only way to query the
values of state variables, such as particle positions and velocities; Context
does not provide methods for accessing them directly.

High performance molecular simulation on GPUs


### Navigation

- Core Objects
- Forces
- Integrators
- Extras

©2015, Stanford University and the Authors.
|
Powered by Sphinx 1.3.1
& Alabaster 0.7.7
|
Page source
